# Understanding how people rationalize victim-blaming and empathy in sex trafficking: a qualitative analysis of 130 narratives

**DOI:** 10.3389/fpsyg.2026.1773545

**Published:** 2026-08-05

**Authors:** Dara Mojtahedi, Kay Lynn Stevens, Adam Austin, Andrea Varsori, Dominic Willmott

**Affiliations:** 1 Department of Social and Psychological Sciences, School of Human and Health Sciences, University of Huddersfield, Huddersfield, United Kingdom; 2 Department of Psychology, Columbia Basin College, Pasco, WA, United States; 3 Department of Criminology, Sociology and Social Policy, School of Social Sciences and Humanities, Loughborough University, Loughborough, United Kingdom; 4 SWPS University, Wrocław, Poland

**Keywords:** exploitation, myths, sex trafficking, sexual violence, victim empathy, victim-blaming

## Abstract

**Introduction:**

Despite the prevalence and severe impact of sex trafficking (ST), research indicates that the crime remains poorly understood by members of the public and professionals in victim-facing roles. Misconceptions about ST, alongside personal biases, have been shown to contribute to victim-blaming attitudes, even when perpetrators have been identified. However, the narrative processes through which such judgements are constructed and justified remain underexplored.

**Methods:**

The present study examined the narrative accounts individuals used to justify both victim-blaming assessments and empathetic concern towards ST victims. Participants (*N* = 130) were presented with a vignette depicting ST and asked to explain and justify their levels of empathetic concern and victim-blaming judgements. An inductive qualitative content analysis identified key themes underpinning these responses.

**Results:**

Most participants expressed empathy towards the victim, with perceptions of coercion, manipulation, and limited agency underpinning supportive responses. However, empathy frequently coexisted with criticism, as many participants simultaneously attributed some degree of responsibility to the victim. Victim-blaming narratives were primarily informed by perceptions of agency, controllability, and personal responsibility, alongside broader misconceptions surrounding exploitation, victimhood, and consent.

**Discussion:**

The findings demonstrate that empathy and victim-blaming are not mutually exclusive but can coexist within the same individual. Attributional judgements regarding victim agency emerged as a central mechanism shaping both supportive and critical evaluations, highlighting the importance of addressing misconceptions surrounding coercive control and victim responsibility to improve criminal justice responses to sex trafficking.

## 1 Introduction

Sex trafficking (ST) is a public health emergency (Anderson et al., 2019; Nemeth and Rizo, 2019), defined as the recruitment, transportation, or subjugation of individuals for commercial sexual exploitation, through coercion or deception (Modern Slavery Act, 2015; U.S. Trafficking Victims Protection Act, 2000). Although there are methodological challenges in determining the true prevalence of ST (Fedina and DeForge, 2017; Franchino-Olsen et al., 2022), it is estimated that 16 million people are trafficked into commercial sex work each year (Ascencion, 2017; Williams, 2018), of which the majority are women and children (Zimmerman and Kiss, 2017). ST victims endure significant harm including emotional, sexual, and physical abuse, with long-lasting impact on their mental and physical health (Lederer and Wetzel, 2014; Reid and Piquero, 2014). Yet despite the pervasive and damaging nature of ST, reports continue to show that many individuals, including professionals in victim-facing roles, perceive victims as being somewhat responsible for their exploitation and abuse (Hartinger-Saunders et al., 2017; Shin et al., 2022; Mojtahedi et al., 2026). Victim-blaming may compound the harms already experienced by survivors of sex trafficking and other forms of sexual violence. Individuals who are subjected to victim-blaming frequently report heightened emotional distress and feelings of invalidation, a phenomenon commonly described as secondary victimisation (Ullman, 2010). Moreover, both exposure to victim-blaming and concerns about being blamed have been shown to discourage victims from seeking emotional, medical, and criminal justice support (Policastro and Payne, 2013; Rajaram and Tidball, 2018). Given the profound harms associated with ST, understanding the factors that shape public and professional responses towards victims is of particular importance. Victim-blaming attitudes have the potential to influence not only public perceptions of victims but also the support, protection, and justice outcomes they receive.

To better understand how victim-blaming is framed and justified, the present study examines the qualitative narratives individuals employ when justifying their empathetic concern and blame attribution in relation to a ST case. The sections that follow outline relevant theoretical models of blame attribution, explore common misconceptions that underpin victim-blaming in cases of ST, and then consider the procedural implications of such perceptions.

### 1.1 Blame attribution frameworks

Numerous socio-cognitive models of blame attribution have been used to explain how situational and dispositional mechanisms inform victim-blaming within sexual violence contexts. The defensive attribution theory (DAT) offers insight into understanding intrinsic motivations that inform victim-blaming. The theory conceptualises victim blame attribution as a mechanism for reducing an observer’s perceived vulnerability to similar offences (Walster, 1966) and consists of two key processes that both predict and are affected by victim-blaming: perceived personal vulnerability and perceived victim similarity (i.e., identifying with the victim’s personal characteristics). Whilst there is agreement that perceived victim similarity influences blame attribution, there are competing explanations on how this relationship operates. On one hand, greater perceived similarity to a victim can foster greater empathy (Paterson et al., 2018) and lead to less blame, partially out of concerns that the observer themselves could be similarly blamed if they were victimised (i.e., defensive blame avoidance; Grubb and Harrower, 2008; Shaw and McMartin, 1977). On the other hand, greater perceived similarity may heighten the observer’s fears of being similarly victimised in the future, which could motivate them to blame the victim as an attempt of defensive harm avoidance (Pinciotti and Orcutt, 2020). The directional relationship between perceived victim similarity and victim blame attribution is likely to vary between individuals depending on which protective motive is more salient (i.e., avoiding blame if victimised oneself versus reducing fears of future victimisation).

The Just world hypothesis (JWH) provides a different motivational explanation for victim-blaming. The theory reflects a biased tendency to perceive the world as inherently just, whereby individuals assume that outcomes people experience are morally deserved (Niemi and Young, 2016). Consequently, when confronted with instances of seemingly innocent suffering, individuals who strongly endorse just-world beliefs may be motivated to generate victim-focused explanations for the offence in order to restore their belief in a fair and orderly world (Van den Bos and Maas, 2009).

The DAT and JWH provide motivational explanations for victim-blaming by highlighting how observers may be driven to protect themselves from perceived vulnerability or preserve beliefs in a fair and orderly world. However, whilst these frameworks help explain why individuals may be motivated to engage in victim-blaming, they offer less insight into how such judgements are constructed and justified when interpreting a specific case of victimisation. Greater insight into these processes can be gained from Weiner’s Attribution–Emotion–Action Model (AEAM) (Weiner, 1986), which explains how causal attributions shape emotional and behavioural responses towards victims. The model posits that individuals interpret events according to their perceived locus of control, stability, and controllability, which subsequently influence emotional reactions and behavioural intentions towards the targeted individual. Particularly relevant to ST victim judgements are perceptions of controllability and locus of control. Incidents perceived to be externally caused and beyond a victim’s control tend to evoke empathy and prosocial responses, whereas incidents perceived to be internally caused and controllable are more likely to evoke anger, criticism, and blame (Yao and Siegel, 2025). Demonstrating the model’s relevance to victim-blaming in sexual violence cases, Sperry and Siegel (2013) observed a relationship between victim responsibility judgements and willingness to help that was mediated by victim sympathy.

Collectively, these frameworks are not mutually exclusive but operate at different levels of explanation. The AEAM focuses on how perceptions of situational characteristics, such as agency, controllability, and responsibility, shape emotional and behavioural responses towards victims. In contrast, DAT and JWH provide motivational accounts of why observers may be inclined to adopt particular interpretations of those situational characteristics. Together, these perspectives suggest that victim-blaming may emerge through an interaction between attributional evaluations of a victim’s circumstances and broader psychological motives that influence how those circumstances are interpreted. However, despite the explanatory value of these frameworks, there remains limited understanding of how these attributional and motivational processes are reflected within individuals’ own narratives when interpreting ST cases. Qualitative exploration of victim-blaming justifications may therefore provide important insight into how theoretical mechanisms identified within these frameworks manifest in real-world evaluations of ST victims.

### 1.2 Victim-blaming misconceptions

The specific narratives that inform victim-blaming in ST cases often draw on inaccurate beliefs about the nature of the offence. A growing body of research demonstrates that both laypersons (Cunningham and Cromer, 2016; Farrell and Pfeffer, 2014; Miller et al., 2023) and professionals in victim-facing roles (Anderson et al., 2017; Hartinger-Saunders et al., 2017; Renzetti et al., 2015; Viergever et al., 2015) continue to endorse ill-informed beliefs about ST—often referred to as *ST myths* (see Mojtahedi, 2025) that perpetuate victim-blaming narratives. These misconceptions can foster victim-blaming both explicitly (i.e., implying that the individual willingly engaged in commercial sex) or implicitly (i.e., suggesting that the victim’s prior choices or behaviours contributed to their exploitation).

One common misconception about ‘real’ victims is the expectation of moral innocence (Vonderhaar and Carmody, 2015). Many fail to recognise that individuals who have broken the law can simultaneously be victims of trafficking (Birks and Gardner, 2019; Buckley, 2009; Santana, 2018). This is particularly problematic in ST cases, as some victims have histories of drug addiction or sex work, often as survival strategies (Koegler et al., 2022; Langton et al., 2022). Past transgressions may also be used to rationalize victimisation through just world beliefs (Van den Bos and Maas, 2009). From this perspective, a victim’s prior behaviour (e.g., drug use) may be perceived as a contributing factor to their exploitation. Although this dynamic has not been explicitly examined in ST contexts, research on other gender-based crimes shows that victims with histories of substance abuse (Grubb and Turner, 2012) or illicit behaviour (Hohl and Stanko, 2015) are more likely to be negatively evaluated.

Victim-blaming narratives also emerge around perceptions of consent, with many victims, including minors (see Hartinger-Saunders et al., 2017), being perceived as having willingly engaged in sex work for personal motives. Perceptions of consent are obfuscated in cases where traffickers offer some compensation to victims (e.g., money or drugs) as a means of manipulation and control (Kara, 2009). Observers often misconstrue compensation as evidence of complicity (Cunningham and Cromer, 2016; Digidiki and Baka, 2020; Farley et al., 2004), which can lead to the rejection of victim status.

Another common victim-blaming narrative centres on the belief that victims choose not to leave their abusers (Mojtahedi, 2025). In reality, ST victims face significant barriers to escape, including fear, coercive control, mistrust of authorities, and other vulnerabilities (Kara, 2009; Langton et al., 2022; Zimmerman et al., 2011). Nevertheless, many overlook these barriers and underestimate the difficulties victims face in leaving their abuse (Houston-Kolnik et al., 2016). Mojtahedi et al. (2026) found that individuals who believed that victims could easily leave their abusers were significantly more likely to engage in victim-blaming when evaluating a ST case.

Many of these misconceptions are particularly relevant in cases where victims are exploited by intimate partners, a pattern that is increasingly recognised as a common pathway into commercial sexual exploitation within Western countries (Walker and Reid, 2024). In some instances, traffickers cultivate or feign romantic relationships with victims as a means of facilitating compliance and control (Kara, 2009). In other cases, sexual exploitation occurs within pre-existing intimate relationships, reflecting the substantial overlap between sex trafficking and intimate partner violence (Roe-Sepowitz et al., 2014; Verhoeven et al., 2015). Research has demonstrated that intimate partners may employ coercion and violence to compel victims into commercial sexual exploitation (Pack et al., 2014; Verhoeven et al., 2015). The victim-blaming narratives observed in ST cases may therefore share similarities with those documented in the wider intimate partner violence and sexual violence literature. Research on intimate partner rape, for example, demonstrates that victims are often denied full victim status because pre-existing relationships are perceived as implying consent or reducing perpetrator culpability (see Lilley et al., 2023, for a review of intimate partner rape myths). Such beliefs may contribute to perceptions that victims exercised greater agency or responsibility for their exploitation than is warranted. Despite these conceptual overlaps, relatively little research has examined how members of the public interpret and evaluate victims of intimate partner commercial sexual exploitation. Greater understanding of these perceptions is therefore needed, particularly given the potential for misconceptions surrounding consent, agency, and coercive control to shape victim-blaming responses.

The acceptance of victim-blaming attitudes, and ST myths more broadly, has been primarily attributed to limited knowledge about the complex nature of the offence (Dando et al., 2019; Digidiki et al., 2016) and to inherent biases toward victim groups (Mojtahedi et al., 2026). Miller et al. (2023) found that participation in an educational intervention presenting factual information about ST reduced misconceptions, including victim-blaming beliefs. These findings suggest that, for some individuals, victim-blaming attitudes may partly stem from a lack of accurate knowledge. However, in another intervention study Mojtahedi et al. (2025) reported that whilst an educational intervention produced a slight reduction in victim-blaming attitudes, educated participants did not differ significantly from controls when attributing victim blame within a specific ST case. While the authors acknowledged a potential ceiling effect, they also noted that victim-blaming judgements may be primarily driven by underlying biases. Supporting this, Mojtahedi et al. (2026) found that hostile sexism was a significantly stronger predictor of victim-blaming than knowledge about the offence.

### 1.3 Implications of victim-blaming for criminal justice practise

The implications of victim-blaming in ST cases are far-reaching and extend into criminal justice practice. At a societal level, acceptance of misconceptions that promote victim-blaming has been shown to reduce public empathy toward victims (Mojtahedi et al., 2026), and diminish support for victim advocacy (Wegrzyn et al., 2023) and victim-focused policies (Clements et al., 2006; Houston-Kolnik et al., 2016).

At a procedural level, victim-blaming attitudes can influence judgements and behaviours among criminal justice professionals and other victim-facing specialists (e.g., healthcare workers). Berishaj et al. (2019) found that police officers often rely on stereotypes and misconceptions when evaluating victims, leading to reduced credibility assessments and inadequate protection. Similarly, Farrell (2014) documented how officers’ preconceptions about victim behaviour shaped investigative practices, sometimes resulting in under-enforcement or case dismissal. Rajaram and Tidball (2018) extended these findings to healthcare professionals, showing that both police interviewers and medical personnel may hold biases—such as labelling survivors as “prostitutes” —that undermine the perceived legitimacy of victim claims and compromise the quality of care provided. Collectively, these studies suggest that professional biases can significantly erode justice and care outcomes.

Further evidence of the influence of victim-blaming attitudes in policing comes from research on other forms of sexual violence. In sexual assault cases, officers are influenced by similar misconceptions about consent, “real rape,” and the prevalence of false allegations (Du Mont et al., 2003; Venema, 2016). Such beliefs perpetuate the dismissal of victimization and the attribution of blame to victims.

Comparable concerns arise in courtroom settings, where juror decision-making is shaped not only by legal evidence but also by extra-legal factors, particularly beliefs about victims (Mojtahedi et al., 2026; Stevens et al., 2021). Birks and Gardner (2019) found that jurors’ perceptions of victim responsibility influenced verdicts, even when unrelated to the legal facts of the case. Similarly, Bornstein and Kleynhans (2019) and Buckley (2009) demonstrated that jurors’ implicit beliefs about the plausibility of victim narratives significantly affect trial outcomes. Conversely, emerging experimental research using mock ST trials suggests that victim-blaming attitudes may not directly determine verdicts (Stevens et al., 2024; Mojtahedi et al., 2025). However, Stevens et al. (2024) reported that participants exhibiting higher victim-blaming attitudes and lower victim empathy expressed greater support for defendants in mock ST cases.

### 1.4 Research gap

Despite theoretical frameworks identifying several cognitive and motivational mechanisms that may contribute to victim-blaming, there remains a notable gap in understanding how these processes are reflected in the narratives individuals use when interpreting ST cases and justifying victim blame. Existing research has primarily relied on quantitative surveys that measure agreement with pre-formulated statements, analyses of victims’ accounts, and, in some cases, examinations of media portrayals. While these approaches have identified common misconceptions and individual differences associated with victim-blaming, they provide limited insight into how individuals actively interpret ST cases and rationalise their blame attributions—or the absence thereof. This limitation constrains our theoretical understanding of the socio-cognitive processes that underpin victim-blaming and empathy, making it difficult to determine why some individuals emphasise victim vulnerability and coercion whereas others focus on agency and responsibility. A qualitative examination of these interpretative narratives is therefore needed to advance understanding of the mechanisms through which victim-blaming attitudes are constructed and maintained and, ultimately, to inform efforts aimed at reducing their endorsement.

The current study adds to our understanding of victim-blaming behaviour by exploring how members of the general public justify victim responsibility assessments when interpreting a ST case. Public attitudes towards sex trafficking victims have broader societal relevance and may influence criminal justice processes indirectly through jury decision-making, public support for victim-focused policies, and the social norms that shape responses to victims (Houston-Kolnik et al., 2016; Stevens et al., 2024). Recruiting a community sample therefore allows the study to capture narrative frameworks that may reflect wider societal perceptions of sex trafficking victims. To explore these attitudes, a qualitative content analysis approach is used examine the narratives that inform victim-blaming, as well as the opposing narratives that support victims. Additionally, a similar analytical approach is used to identify key themes that inform affective concerns towards ST victims, as well as non-empathetic responses.

## 2 Methods

### 2.1 Participants

Participants from the UK were recruited through an online crowdsourcing platform (Prolific) and were compensated the equivalent of $2 for participation. The platform’s demographic pre-screening function was used to ensure that participants from a relatively balanced range of political orientations were recruited, owing to past research demonstrating the victim-blaming in ST cases is more prevalent among right-wing individuals (Mojtahedi et al., 2026). One hundred and thirty participants (84 females) with a mean age of 44 (Std dev = 13.83), completed the experiment (all participants passed the attention questions [recalling key details from the vignette]). Given the disproportionate representation of female participants within the sample, exploratory analyses were conducted to determine whether overall empathy and blame judgements differed by gender (see Section 3).

### 2.2 Materials and procedure

A ST vignette was developed in consultation with an external professional who was involved in the protection of ST victims within Northwest England. Initially, the authors constructed a vignette based on a real ST case whereby a young female (*Stephanie*) was coerced by her partner (*Ryan*) into commercial sexual exploitation. Following consultation with the external professional, the vignette was revised to more accurately reflect ST cases in the UK. The consultation reaffirmed the researchers’ decision to feature a domestic scenario, after it was confirmed that exploitation frequently occurs within pre-existing romantic relationships, where emotional attachment, dependency, and coercive control are used to facilitate compliance and inhibit disclosure or escape. The revisions also included describing the victim’s drug use; and highlighting the victim’s difficulties in leaving her partner.

The finalised vignette describes a case where Stephanie, a 23-year-old woman reported that her former partner, Ryan, sexually exploited her for financial gain over the course of their relationship. After initially meeting Ryan as a vulnerable teenager, Stephanie later reconnected with him in adulthood and moved into his home, where he appeared caring and supportive. Several months into the relationship, Ryan began coercing her into having sex with other men in exchange for money, using emotional pressure, guilt, repeated persuasion, and later threats of homelessness and withdrawal of affection. The exploitation escalated in frequency and severity, alongside Stephanie’s increased reliance on cocaine supplied by Ryan as a coping mechanism. Stephanie eventually fled to a women’s shelter and reported the abuse to the police, leading to an ongoing investigation into Ryan’s actions (for full vignette, see Supplementary materials).

Participants were asked to carefully read the vignette and were informed that they would be asked questions about the case. After reading the vignette, participants were first asked to justify whether or not they felt empathy towards the vict using an open-ended response box; they were encouraged to start their response with “I [feel/do not feel] sorry/empathy for Stephanie because…” Next, participants were asked in a similar format to justify whether or not they felt that Stephanie was partly responsible for everything that happened; they were encouraged to start their response with “I [think/do not think] Stephanie is partly responsible for the incidents because…” The study received ethical approval from the lead author’s institutional Research Ethics and Integrity Committee.

### 2.3 Content analysis

Participants provided short but substantive responses to two open-ended questions. Responses to the empathetic justification question averaged 32.88 words (SD = 20.58) or 1.74 sentences (SD = 1.14), while responses to the victim blame attribution question [“In the box below, please justify whether or not you feel that Stephanie is partly responsible for everything that happened (e.g., I think/do not think Stephanie is partly responsible for the incidents because…”)] averaged 26.91 words (SD = 19.46) or 1.47 sentences (SD = 0.81). Given the brevity and conceptual nature of these responses, an inductive qualitative content analysis approach was employed, which is recommended for short open-ended survey data (Hsieh and Shannon, 2005; Züll, 2016). An inductive approach was chosen over guided content analysis because the analysis was exploratory and sought to derive narrative categories directly from the data rather than from pre-defined theories. By doing so, the researchers mitigated the risks of omitting key factors that may not have aligned with past victim-blaming frameworks. Despite some awareness of relevant theoretical frameworks, coders were instructed to derive coding categories directly from the data rather than informed by pre-existing models.

The analysis focused on identifying conceptual arguments rather than counting lexical items. Latent coding was applied (Kleinheksel et al., 2020) to label the underpinning justifications that informed responses. Semantically related phrases (e.g., “threatened,” “coerced”) were grouped under broader conceptual codes (e.g., “forced to act”). This allowed systematic quantification of the presence of specific arguments or positions within each response.

An initial coding scheme was devised to capture five broad categories for each question: (1) main judgment on the topic at hand (affective concern/responsibility), (2) reasons for empathy/responsibility, (3) reasons for lack of empathy/responsibility, (4) broader comments about the victim, and (5) broader comments beyond the victim. A pilot analysis of 20% of responses was conducted using an inductive exploratory approach to identify these five categories, as well as key phrases within them. Following established guidelines (Elo and Kyngäs, 2008; Schreier, 2012), these phrases were then grouped into concept-driven codes within each category.

During piloting, it became evident that some participants provided conditional or partial judgements rather than categorical responses. Consequently, coding options for empathy and responsibility judgements were expanded to include partial, conditional, unclear, and unstated responses. The codebook was iteratively refined to clarify definitions and grouping rules (e.g., “forced to act” included phrases such as “coerced,” “forced,” “threatened”). Revisions included merging conceptually similar codes (e.g., “manipulated” into “used”) and adding new codes (e.g., “vulnerability”). The final codebook comprised 32 codes, presented in Table 1.

**Table 1:** Qualitative content analysis codes and frequencies.

Code	*N (%)*
Victim empathy response codes	
*Outright judgement*	
Empathetic	104 (80%)
Empathetic but critical	10 (7.69%)
Non-empathetic	5 (3.85%)
Unstated	8 (6.15%)
Unclear	3 (2.31%)
*Empathetic justifications*	
Victim was used	30 (23.08%)
Groomed	15 (11.54%)
Betrayal of trust	14 (10.77%)
Adverse childhood	21 (16.5%)
Entrapment	18 (13.85%)
Condemnation of abuse	13 (10%)
*Justifications for lack of empathy*	
Consented to partner	3 (2.31%)
Could have refused	5 (3.85%)
Adult status	4 (3.08%)
*Broader issues (both responses)*	
Prevalence	5 (3.85%)
Protection from dating violence	1 (0.77%)
Victim blame attribution response codes	
*Outright judgement*	
No responsibility	76 (58.46%)
Minimal responsibility	5 (3.85%)
Partial victim blame	31 (23.85)
Explicit victim blame	9 (6.92%)
Unstated	3 (2.31%)
Unclear	6 (4.62%)
*Justifications for no responsibility*	
Victim was used	53 (40.77%)
Victim was forced	24 (18.46%)
Victim vulnerabilities	41 (31.54%)
Unspecific vulnerability	7 (5.38%)
Young age	18 (13.85%)
Upbringing	10 (7.69%)
Lack of support	6 (4.62%)
*Victim blaming justifications*	
Could have left/sought support	28 (21.54%)
Rejected initial request	10 (7.69%)
Complicit	8 (6.15%)
She had a choice	15 (11.54%)

All responses were coded for the presence (1) or absence (0) of each conceptual code. Two researchers independently coded all 130 responses using the final codebook. Inter-rater reliability was assessed using Cohen’s Kappa (*κ*), with values ranging from 0.892 to 1.00, indicating substantial to almost perfect agreement (Landis and Koch, 1977; McHugh, 2012). For empathy justification responses, discrepancies were limited to three codes and varied by one or two counts. For victim blame attribution responses, discrepancies were minor with almost all differences in code frequencies varying by less than four counts. The only exception was for the scoring of *victim was used* which was originally labelled as *victim was manipulated* (*n* = 48 vs. 53): The first coder had omitted five responses due to not feeling that the theme of manipulation was explicitly defined, however the second coder acknowledged cases where it was acknowledged that the victim had been used for commercial sexual profit; following the settlement of this discrepancy, the label was also changed. Discrepancies were thus resolved through discussion, and the coding frame was refined accordingly.

## 3 Results

Findings are separated into two sections, first examining participants’ justifications for empathetic concern (or lack of) followed by justifications for victim blame attributions. Each section commences by outlining the prevalence of different outright judgements, followed by justifications for and against victim empathy/blaming. A list of all relevant codes, and their prevalence rates, are presented in Table 1. Due to the sample being disproportionally female, chi-square analyses were conducted to determine if the prevalence of outright judgements were swayed by gender distribution (gender distribution for these responses are presented in Table 2).

**Table 2:** Cross-tabulated gender distribution for empathy judgement and blame attribution.

Code	Gender distributions (% within gender group)^a^	
	Male (*n* = 45)	Female (*n* = 84)
Empathy judgement		
Empathetic	32 (71.2%)	72 (85.7%)
Empathetic but critical	5 (11.1%)	5 (6%)
Apathetic	0%	4 (4.8%)
Unstated/unclear^b^	8 (17.8%)	3 (3.6%)
Blame attribution		
No responsibility	21 (46.7%)	53 (63.1%)
Minimal responsibility	3 (6.7%)	3 (3.6%)
Partial victim blame	13 (28.9%)	17 (20.2%)
Explicit victim blame	3 (6.7%)	6 (7.1%)
Unstated/unclear^b^	5 (11.1%)	5 (6%)

a One participant removed from comparison due to undisclosed gender.

b Due to low cell counts and conceptual similarity, unclear and unstated responses were merged for analysis.

Initial coding criteria included two supplementary categories designed to capture broader victim-related themes and wider contextual themes. In practice, the content identified within these categories was either integrated into existing core themes (e.g., stating that the victim should have been stronger was merged into the *preventative controllability* theme; and *condemnation of abuser* was combined with *empathetic concern*) or omitted from further analysis because occurrences were minimal (i.e., *n* = 1) and offered little substantive contribution to the study’s aims. The more meaningful of the minimal codes are however still included where relevant in this section.

### 3.1 Empathetic concern

Most participants reported feeling empathy for the victim (*n* = 114, 87.69%), however 10 of these participants disclosed an “empathetic but critical” disposition. Only five (3.85%) respondents explicitly disclosed a lack of empathy towards the victim, and the remaining responses were either unstated (*n* = 8, 6.06%) or unclear (*n* = 3, 2.3%) in response to the empathetic concern question. Chi-square test revealed a significant difference in outright empathy judgements between male and female participants (χ^2^ (3) = 10.86, *p* = 0.013). Cross-tabulated percentages revealed that none of the non-empathetic responses were from male participants; male participants were also overrepresented among those providing unstated or unclear responses.

As will become apparent within the results section, there were some notable crossovers in justification themes informing both empathetic judgements and blame attributions, this typically surrounded perceptions of internal and external causes of the exploitation.

#### 3.1.1 Empathetic justifications

As stated, most participants felt empathy towards the victim with some (*n =* 17, 13.08%) exclaiming explicitly high levels of empathy (e.g., *“I feel deep empathy”*) or sympathy (“*I feel very sorry for Stephanie,” “I feel terribly sorry”*) towards the victim. P10 (f) stated *“I pray that she gets support and gets looked after,”* whilst P11 (m) believed that empathy was the only possible response, stating *“I cannot imagine anyone who would not feel empathy for Stephanie.* Six overarching themes were brought up y participants to justify their empathetic concern for the victim, with many participants reporting multiple reasons.

##### 3.1.1.1 Victim was used

The most stated reason for victim empathy was acknowledgment that she had been used for commercial exploitation (*n* = 30, 23.08%). Some went further acknowledging that their empathetic concern came from understanding that the victim did not want to engage in the sex work but was manipulated into it by the perpetrator—vocalising a perceived sense of injustice. The victim’s need for love and care was a recurrent theme brought up in various responses under different contexts (including here). Some saw her need for love as a vulnerability, a justification for her perceived compliance, or as a reason to condemn the perpetrator for abusing this vulnerability.

*P32 (f)“…[perpetrator] took advantage of her vulnerability and need for love and protection.”*

*P43 (m) “I do feel for her as she has been manipulated into doing things she clearly did not want to do. It feels very much like emotional blackmail.”*

Additionally, two (1.54%) participants stated that they were able to relate to the victim because of experiencing similar abuse (personally and indirectly through victim support).

##### 3.1.1.2 Groomed

Eleven participants (8.46%) drew on the fact that they believed the victim had been groomed, when justifying their empathetic concern. Particularly, participants pointed out the significant power dynamic resulting from the age disparity and acknowledged the limited agency the victim had in preventing her exploitation from an early age.

*P73 (f) “She had very little power in the relationship and was groomed from a young age to view [perpetrator] as a good person.”*

*P6 (f) “Although Stephanie was a young adult by the time her sexual relationship began with [perpetrator], a) he was much older than her, and b) buying her alcohol at 15, when he was 30 years old, and chatting to her can be considered grooming.”*

Two participants (1.54%) were able to empathise with the victim due to having greater awareness of exploitation through work or personal experiences, demonstrating that greater knowledge and experiential closeness to victims allows for greater appreciation towards their hardship. In one case, the participant was able to empathise with the victim due to having worked with victims of child grooming.

*P127 (f) I feel true empathy for Stephanie as she was groomed and manipulated into a situation. I work with vulnerable Children and Adults this sort of thing has happened to and it happens more often than people think.*

##### 3.1.1.3 Betrayal of trust

Participants (*n* = 14, 10.77%) also cited the offender’s betrayal of the victim’s trust as justification for their empathetic concern towards the victim. Participants often connected this with the love and affection that the victim had for her abuser. The fact that the perpetrator had pretended to engage in a romantic relationship with the victim in order to abuse her was clearly seen as a seriously aggravating circumstance, worthy of further empathy for the victim and additional condemnation for the abuser.

*P50 (f) “She trusted the one person she thought loved her and she was exploited as a result.”*

*P71 (f) “I feel empathy because she genuinely loved and cared for this man who took advantage of her and basically forced her to sell her body to men.”*

##### 3.1.1.4 Adverse childhood

Participants (*n* = 21, 16.15%) also reported feeling empathy for the victim due to her adverse childhood, a factor that many participants believed made her more vulnerable to sexual exploitation as an adult. In a few cases (*n* = 3, 2.31%), this was expressed in terms of an absence of guardians, with participants indicating that the parents have been ‘neglectful’ and ‘not responsible’ and that the victim had received ‘no guidance’. Similarly to the discussion of grooming, participants talked about these early experiences as though they placed the victim on an early trajectory towards future victimisation.

*P69 (f)* “*I feel bad for Stephanie and have empathy for her because it seems that she did not grow up in a loving supportive home and because of this it made her vulnerable to being taken advantage of and abused.”*

*P65 (f) “I feel sorry for Stephanie because it’s clear her upbringing, coming from a poor home with neglectful parents, influenced her to run away on occasions to see [perpetrator].”*

##### 3.1.1.5 Entrapment

Many participants (*n* = 18, 13.85%) justified their empathy towards the victim by commenting on the victim’s difficulties in leaving her partner/perpetrator, describing her ordeal as a form of entrapment that was strategically orchestrated by the perpetrator. Particularly, empathy appeared to stem from the victim’s loss of agency. Two participants (1.54%) also acknowledged the ease of being manipulated and difficulties in escaping as common challenges faced by such victims.

*P117 (f) “I feel deep empathy for Stephanie as its not as easy as just leaving for some people who are being controlled like this.”*

*P118 (f) “He lured her in, gained her affection, trust. He then used fear and [drug] addiction to force his will on her. She was trapped.”*

##### 3.1.1.6 Condemnation of abuse

Participants (*n* = 13, 10%) explained their empathetic reactions through condemning the abuse, highlighting the abhorrent nature of the exploitation as a cause for concern towards the victim. Condemnation was characterised by the use of emotionally valanced descriptors, further highlighting the abusive behaviour as abnormal and wrong. Many participants also referred the abuse as an (atrocious) experience that the victim ‘went through’, acknowledging the longevity of the abuse.

*P121 (m) “I feel sorry for her because this is truly disgusting and horrific for a young woman to go through.”*

*P67 (m) “Her ordeal sounds horrific, and no one should have to go through what she has.”*

Some of these participants (*n* = 6, 4.62%) expressed anger more explicitly, by disclosing their reaction to the perpetrator or through capitalisation of the text.

*P120 (f) “I feel angry that [perpetrator] has treated her this way.”*

Perhaps the most dramatic condemnation came from a male participant who believed more drastic measures were required to resolve ST. This participant framed ST as a male-perpetrated offence that women needed protecting from.

*P121 (m) “if it were up to me, ALL MEN INVOLVED WOULD BE PUT TO DEATH if found guilty, to make the world a better place and protect women from this.”*

#### 3.1.2 Justifications for lack of empathy

Only five participants (3.85%) reported not feeling empathy towards the victim, although one of these participants still acknowledged the victim’s ordeal as ‘terrible’. Reasons for not empathising with the victim were less nuanced, with participants providing three key reasons, all relating to some form of victim accountability. Very few participants acknowledged or discussed whether the victim has suffered.

##### 3.1.2.1 Consented to partner

Three out of the five participants showed no empathy towards the victim due to believing that she willingly engaged with her partner/perpetrator’s behaviour, with some also attributing blame to her initial engagement with the perpetrator at 15. Here participants were not considerate towards the victim’s suggestibility or impaired judgement at such as young age.

*P14 (f)“i feel no empathy for someone in her situation as she put herself in it at 15 years old.”*

*P58 (undisclosed gender) “I do not feel as much empathy towards Stephanie as I think a 15 year old should know someone giving them drugs in a dodgy pub is morally wrong. …. She obviously knew it was wrong to sleep with other men for money. That should have been the red line and she should have walked away, but she did not. She was obviously a weak character and a drug user.”*

Participant 58’s framing of the behaviour as ‘sleeping with other men for money’ further illustrated their perception of consent. Also, along with other participants, this individua used the perceived immorality of the victim’s behaviours (e.g., substance use and perceived prostitution) to void empathy whist ignoring the harm the victim suffered.

##### 3.1.2.2 Could have refused

All five unempathetic participants (3.85%) believed that the victim had the opportunity to refuse their partner’s request in engaging in the sex work during the first incident. Only one participant mentioned that the victim should have reported the incident to the police. Perceptions around the victim’s level of inaction were used to suggest some involvement and therefor, rejection of empathetic concern. Many participants framed the abuse as having been “allowed” by the victim, further articulating such beliefs.

*P80 (f) “I do not feel much empathy for Stephanie because she allowed herself to be used, she should have left him at the start. It is a bad situation which I would not like, but she should have taken action sooner.”*

*P98 (f) “I do not feel sorry for her. I feel that the first time was when she should have put her foot down because if she had done it once, then [perpetrator/partner] knew he could manipulate her to carry on.”*

##### 3.1.2.3 Adult status

Four out of the five participants also pointed out the victim’s age during her exploitation, explaining that their lack of empathy towards her was due to the fact that she was an adult who was capable of refusing her partner/perpetrator’s requests. Two of the participants contrasted the earlier age in which the victim met the perpetrator to the age in which the exploitation started. Another participant indicated instead a connection between age, morals, and responsibility:

*P33 (f) “I do not feel sorry for Stephanie as she was 20 at the time and not a child any more.”*

*P58 (undisclosed gender) “By 20, her morals and individual responsibility should have been stronger. She obviously knew it was wrong to sleep with other men for money.”*

#### 3.1.3 Empathetic but critical response

As stated previously, some participants reported feeling empathy for the victim but provided an ‘empathetic but critical’ response when justifying their empathetic responses (i.e., similar criticism was reported despite feeling some empathy). The motivations for a more critical response were varied: they include the three mentioned in the previous subsection, but a few participants also expressed doubt regarding potential pro-victim bias in the vignette or incredulity for the fact of falling victim of such emotional manipulation.

*P27 (m) “I do feel empathy for her, however she should have said no to start with or left earlier.”*

*P23 (f) “I do have empathy and she was gullible and trusting… How can anyone believe someone loves them if they are being prostituted.”*

Participant 23’s use of “prostituted” as opposed to victimisation terms (e.g., exploited) may also highlight perceptions and framing of the incident as being consensual sex work rather than abuse. A male participant also questioned whether the empathy given to the victim was influenced by the fact that she was a female, citing double standards in the way we perceive cases of alleged sexual violence and victim empathy. This suggests that empathetic reactions to victims of sexual exploitation may be influenced by gender biases.

*P112 (m)* “Would people have as much pity if the roles were reversed?”

#### 3.1.4 Unstated and unclear responses

Of the remaining participants, eight (6.06%) offered some interpretation of the event, such as acknowledging the harm done, attributing responsibility, or claiming that this type of events is frequent. However, they refrained from specifying whether empathy was felt or not.

*P46 (m) “She clearly loved [perpetrator] and initially started having sex with others to maintain his affection and help their relationship financially, this led to drug addiction and further exploitation.*

*P38 (m) “It was a bad situation she got herself in.”*

A further three participants (2.31%) were unclear in their stance, possibly reflecting uncertainty. For one participant, this was due to simultaneously finding both the victim and perpetrator culpable. Interestingly, the term “allow” was used again suggesting a possible conflict between acknowledging suffering but also framing the victim as accountable.

*P109 (m) “On face value Stephanie has been mistreated and taken advantage of,but did allow herself to some extent to be abused.”*

Another participant acknowledged difficulties in determining the “truth” due to not hearing the perpetrator’s side of the story – this was also acknowledged by two participants who had expressed empathy towards the victim but demonstrated a sense of scepticism. Implications within these responses could also suggest that some participants have reasons to question the victim’s believability – a common reaction observed among jurors in sexual violence cases.

*P91 (m) “I try to look at things objectively, and there always two sides to a story and it is difficult to ascertain the truth.”*

*P121 (m) “However, both sides of the story need to be heard for me to fully support her. I cannot make up my mind about someone based on someone else’s testimony.”*

### 3.2 Victim blame attribution

Coding of blame attribution judgements identified five categories of victim blame: No responsibility (*n* = 76, 58.46%), minimal responsibility (*n* = 5, 3.84%), partial victim-blaming (*n* = 31, 23.84%), explicit victim-blaming (*n* = 9, 6.92%), unstated (*n* = 3, 2.31%), and unclear (*n* = 6, 4.62%). Chi-square test revealed no significant gender differences in outright victim blame attribution judgements (χ^2^ (5) = 3.94, *p* = 0.414).

#### 3.2.1 Justifications for no responsibility

The majority of participants placed no responsibility on the victim, with two participants exclaiming this point (e.g., “*she is totally not responsible,” “I do not think Stephanie is responsible at all”*). Key justifications for no responsibility centred around the belief that the victim was used or forced into the sex work, as well as the acknowledgement of the victim’s vulnerabilities (age, upbringing and lack of support).

##### 3.2.1.1 Victim was used

The most common justification for removing victim responsibility was the perception that the victim had been used by the perpetrator (*n =* 53, 40.77%). Participants focussed their justifications on detailing the role that the perpetrator played in manipulating the victim.

*P67 (m) “I do not think Stephanie is partly responsible for what happened to her. The case details point to [perpetrator] manipulative and controlling behaviour. He used kindness and affection to gain her trust, then shifted to guilt trips and veiled threats to coerce her into sex work.”*

Some female participants also spoke more generally about the ease of manipulation within relationships, demonstrating a sense of understanding towards the risks of coercive control and the difficulties in escaping it.

*P130 (f) “People do crazy things when in love and they do not always see things as you’d expect.”*

*P85 (f) “if you are being asked to do something by a person who you love deeply is difficult.”*

*P18 (f) “I do not believe Stephanie is responsible, it is extremely difficult to leave an abusive relationship, especially when you have no other support system.”*

##### 3.2.1.2 Victim was forced

Some participants (*n* = 24, 18.46%) went further arguing that the victim was not just used but forced into the sexual activity, implying that the opportunity to dissent was not just difficult but impossible. The use of fear and drugs were frequently mentioned as mechanisms for complete control.

*P108 (m) “[perpetrator] exploited her vulnerable state, used emotional manipulation, threats, and control over her substance use to force her into situations she did not want to be in. Her initial consent was obtained through persistent pressure and emotional blackmail, making it clear that she was not acting of her own free will. The power dynamics in their relationship were heavily skewed in [perpetrator] favour, leaving Stephanie with little autonomy…”*

*P41 (f) “I do not think Stephanie is partly responsible in this incident she was being threatened and forced to do things against her will.”*

*P76 (f) “She was groomed and coerced into these acts by someone she thought loved her, he provided drugs to get her hooked, then would only provide them when she did what he said. It’s coercive control.”*

Five (3.85%) of these participants further evidenced their beliefs that the victim had been forced into sexual exploitation by acknowledging her earlier attempts to refuse the sex work. They stressed that the victim had expressed her dissent initially and had then been threatened; these are clearly seen as important in voiding her later consent and thus her responsibility.

*P20 (f) “I do not feel that Stephanie is responsible. She said she did not want to do these things, but was coerced into doing so.”*

*P36 (m) “I do not think Stephanie is partly responsible because she was forced into it after repeatedly saying no.”*

*P127 (f) “Stephanie was clearly groomed and continually told [perpetrator] she did not want to do it.”*

##### 3.2.1.3 Victim vulnerabilities

Participants highlighted the victim’s vulnerabilities 41 times, using this factor as a reason to remove responsibility from them. Whilst 7 participants (5.38%) did not specify what these vulnerabilities were, the remaining participants specified vulnerabilities relating to the victim’s young age, upbringing and general lack of support.

*Young age*: Participants (*n* = 18, 13.84%) questioned the cognitive and emotional maturity of a young adult in making informed decisions, whilst others highlighted how the age gap created a power dynamic that allowed the perpetrator to control the victim. These justifications presented the victim as a vulnerable individual susceptible to manipulation.

*P94 (f) “I do not think she is responsible as she was vulnerable and groomed by a much older man.”*

*P106 (f) “I do not think that Stephanie is responsible, she is at an age where her brain is still developing. She has landed in an abusive situation and did what she could in that time to protect herself.”*

Here, too, a few of the respondents highlighted the victim’s age when she first met the perpetrator (15 years old). Whenever this is stressed, it is often done so in connection with the idea of grooming. Even though the exploitation started only much later in the vignette, this detail is clearly very striking for some of the participants.

*Upbringing*: The victim’s adverse upbringing was also used by some participants (*n* = 10, 7.69%) to explain why she was drawn to and remained attached to her exploitative perpetrator. Some participants expanded on this explanation, believing that the absence of a healthy parental relationship made her seek the attention of other adults. Although somewhat speculative, these interpretations represent acknowledgement of the victim’s vulnerability.

*P50 (f) “I do not think Stephanie is responsible as she was out drinking at a young age as she had no support from family and no role model. She chose an older man with perhaps the idea he would help her.”*

*P37 (m) “Stephanie obviously had a bad family life and a poor upbringing. She had very few few friends to turn to.”*

*Lack of support*: Her apparent lack of support—in terms of friends, family, or even a place where to live—was also used by five participants (3.85%) to explain her difficulties in leaving the perpetrator, believing that she had limited options (both in terms of emotional support and resources) outside her exploitative circumstances.

*P18 (f) “I do not believe Stephanie is responsible, it is extremely difficult to leave an abusive relationship, especially when you have no other support system.”*

*P36 (m) “She could have run away and not do it but then she would have been homeless with nowhere to go.”*

#### 3.2.2 Victim-blaming justifications

Explicit victim-blaming was justified by only nine participants (6.92%) who believed that the victim was responsible for her exploitation. However, a further 31 participants (23.85%) believed the victim was partially responsible, believing that whilst she was a victim of a crime her actions, or inaction, contributed to her exploitation. The justifications provided for complete and partial victim-blaming similarly drew on beliefs around victim autonomy during sexual exploitation.

##### 3.2.2.1 Victim could have left or sought support

The most common justification for victim-blaming was the belief that the victim could have prevented the ongoing exploitation by leaving the perpetrator or seeking out support (*n* = 28, 21.54%). Participant 21 also added additional instances where the abuse could have been prevented, suggesting that the victim had multiple opportunities to avoid the sexual exploitation, further compounding blame attribution.

*P70 (m) “I feel that she could have left quite easily.”*

*P21 (f) “Stephanie is partly responsible only because she could have made the decision to seek help sooner or avoid drugs and alcohol.”*

##### 3.2.2.2 Victim could have rejected the initial request

Whilst many participants acknowledged that the victim had ended up in a situation that was difficult to escape, some participants (*n* = 10, 7.69%) believed that she could and should have rejected her partner’s initial requests of sex work. This contrasts with responses from participants who attributed no responsibility to the victim, several of whom highlighted her initial refusal as evidence that she did not consent to the exploitation (see Section 3.2.1.2).

*P46 (m) “Stephanie could have left the relationship much earlier and refused to have sex, it would have been difficult but entirely possible.”*

*P8 (f) “I think Stephanie is partly responsible because the first time this occurred she was in a public place where she could of sought help.”*

Three participants (2.31%) also believed that the victim could have initially rejected her partner’s demands if she was stronger, however only two (male) participants used this to attribute some responsibility onto the victim. Participant 93 stating that the victim could have been stronger suggests that responsibility was partly attributed to the victim’s perceived failure to resist or prevent the abuse. The other (female) participant acknowledged the victim’s lack of strength but used it to remove responsibility.

*P93 (m) “She is slightly responsible, the drugs may have more influence than is being implied and she could have been stronger at the first hint of this.”*

*P61 (f) “I think Stephanie was left with no choice but to go along with her partners demands, she’s not strong enough to stick to her initial refusal.”*

##### 3.2.2.3 Complicit

Some participants (*n* = 8, 6.15%) believed that the victim demonstrated some degree of complicity towards the sex work. Verbs such as “submitted” and “agreed for sex” were used to denote voluntary behaviour, and one participant highlighted that the victim received some money, possibly to insinuate a motive.

*P27 (m) “I do think she is [partially] responsible as she initially submitted to his demands to have sex for cash and then continued to do it.”*

*P15 (f) “She is partially responsible for what happened because she agreed for sex with other men.”*

##### 3.2.2.4 She had a choice

The concept of “choice” was repeatedly invoked (*n* = 15, 11.54%) to reframe the situation from one of coercion or victimisation to one characterised by agency. In doing so, participants implicitly positioned the victim as accountable for her circumstances. This also featured in some cases where participants acknowledged that the victim was not entirely responsible but continued to contend that some of her actions contributed to her victimisation. As demonstrated by participant 23’s use of “made the wrong choice,” many responses contended that the exploitation was the result of the victim’s errors.

*P33 (f) “she has her own mind and should have walked away from the start.”*

*P23 (f) “Not responsible as such but made the wrong choice and found herself trapped.”*

One participant also suggested that the victim’s recovery following the abuse was dependant on her ability to acknowledge her own faults.

*P6 (f) “There were opportunities for her to make different choices. Recognising where she could have made better choices will aid in her recovery and future life path…”*

Two participants (1.54%) further extended this narrative by asserting that all individuals possess the capacity to choose their actions, thereby constructing a moral comparison between the victim and non-victims based on individual decision-making and personal responsibility.

*P14 (f) “Yes, everybody has a choice.”*

*P88 (m) “Yes, I think she is partly responsible for what happened, because everyone [does] have a choice.”*

#### 3.2.3 Minimal responsibility

The five responses (3.85%) that were categorised as *minimal responsibility* reflected judgements where participants acknowledged that victim agency could not be completely ruled out but were negligible. One participant explicitly acknowledge that such minimal blame should not be acknowledged in court. However, none of these cases provided reasons why some responsibility was placed onto the victim.

*P113 (f) “I think Stephanie is only barely responsible for her situation.”*

*P1 (m) “Objectively it may be possible to give her a very small part of the responsibility, but this is negligible and certainly would not deserve mention in court.”*

#### 3.2.4 Unstated or unclear

Nine participants (6.92%) were unclear around whether they perceived the victim as having some responsibility in her ordeal. Three participants provided some commentary on the situation, often highlighting the victim’s errors, without answering the question.

*P98 (f) “She hung on to [perpetrator] when he clearly did not value, love or respect her.”*

*P123 (f) “She was in a confusing situation.”*

The other six responses acknowledged arguments for and against victim-blaming (aligning with the previous identified justification themes) but emphasised some uncertainty around whether they believed the victim was responsible.

*P69 (f) “I think most people have agency and therefore are in some way responsible for their actions and decisions. However, I think because of the way that she was raised Stephanie wasn’t fully able to understand what she was getting into and she was taken advantage of by someone older and most likely more experienced.”*

*P115 (f) “I think everyone has autonomy; however she was put in a very difficult situation with minimal support from her life.”*

### 3.3 Broader issues

In explaining their empathy and perceptions of responsibility some participants touched up on beliefs surrounding wider issues around sexual violence. Five participants (3.85%) commented on the wider prevalence of sexual exploitation that has prevails in modern society.

*P96 (m) “I know this sort of thing is a lot more common than you would expect in this day and age. It seems to be very easy for men to manipulate women into doing these sort of things.”*

*P127 (f) “…it happens more often than people think.”*

This knowledge led one participant into suggest that more needs to be to protect individuals from dating violence.

*P53 (m) “I think we need to educate young people about healthy relationships and to help them see signs of abuse as it comes down to experience.”*

## 4 Discussion

To the authors’ knowledge, this is the first study to qualitatively examine the narrative frameworks through which individuals justify both concern and blame towards victims of ST. Perceived victim agency emerged as a central mechanism underpinning both empathic and victim-blaming evaluations, with responses being consistently centred on perceptions of victim agency, controllability, and responsibility. However, participants frequently interpreted these factors differently, drawing upon the same case information to construct contrasting evaluations of the victim.

Overall, the findings indicate a predominance of empathetic concern and minimal outright victim blaming, which may appear inconsistent with arguments that there are widespread victim-blaming attitudes towards victims of sexual exploitation. However, it should be noted that the vignette depicted a relatively severe case involving various mechanisms of exploitation (e.g., grooming, coercive control, and threatening behaviour) that may have increased empathic responding. Furthermore, many participants simultaneously expressed empathy for the victim while endorsing narratives that attributed some degree of blame, indicating that affective concern and blame attribution can coexist within the same individual. Consequently, the significance of these findings lies not in the prevalence of overt victim-blaming, but in the persistence of victim-blaming narratives within a largely empathetic sample.

Taken together, these findings make two key contributions. First, they demonstrate that evaluations of ST victims are shaped not simply by the presence of cues relating to victim agency, controllability, and responsibility, but by how these shared cues are interpreted, allowing the same evidence to support either empathic or victim-blaming narratives. Second, the findings challenge the assumption that empathy and victim-blaming represent opposing evaluative positions. Instead, many participants simultaneously expressed concern for the victim while attributing some degree of responsibility, leading us to propose the concept of *conditional empathy*. This concept captures the finding that empathy does not necessarily preclude blame attribution; rather, the extent to which empathy mitigates perceived victim responsibility appears to depend on the extent to which participants perceive the victim’s actions as consistent with their expectations of victims of sexual exploitation. These contributions are considered in turn below.

### 4.1 Perceived agency as a mechanism underpinning empathic and victim-blaming evaluations

A central contribution of the present study is the identification of perceived victim agency as the primary mechanism associated with both empathetic and blame-related judgements. Although empathy and blame are often conceptualised as distinct processes, the findings suggest that they are co-produced through shared attributional reasoning. Specifically, participants’ justifications consistently revolved around assessments of the victim’s control, autonomy, and capacity to prevent the abuse.

This pattern is compatible with Weiner’s (1986) Attribution–Emotion–Action Model (AEAM), which posits that perceptions of controllability and locus of control shape emotional responses such as sympathy or anger. Participants appeared to draw upon two competing attributional frameworks. On one hand, externally oriented explanations emphasised coercion, grooming, and structural vulnerability, thereby framing the incident as difficult to prevent, reducing perceived agency and eliciting empathy. On the other, internally oriented explanations framed the victim’s actions as voluntary and the abuse as preventable, often invoking notions of choice, morality, and personal responsibility, which in turn justified blame and reduced empathy.

Notably, these attributional processes were not mutually exclusive. A subset of participants adopted hybrid positions, reflected in “empathetic but critical” responses, wherein external constraints were acknowledged alongside residual expectations of personal responsibility. This finding is consistent with attributional research demonstrating that perceived preventability can sustain blame even in the presence of external causal factors (Alicke, 2000; Shaver, 1985). Furthermore, the AEAM framework suggests that controllability judgements (i.e., the extent to which an outcome is perceived as preventable or avoidable) may exert greater influence than locus of control (i.e., whether the cause of an outcome is perceived as internal or external to the individual) in shaping moral evaluations (Weiner, 1986), which would explain why many participants acknowledged and empathised with the victim’s suffering whilst still maintaining that she had some responsibility for failing to prevent the exploitation.

The data revealed that identical case features could be interpreted in divergent ways to support opposing judgements. For example, the victim’s age at initial contact (15) was construed by some as evidence of vulnerability and grooming, whilst others interpreted it as indicative of sufficient maturity and responsibility. Similarly, the victim’s initial resistance was either foregrounded as evidence of her objection or dismissed as either inadequate or non-existent. These contrasting interpretations demonstrate how the same case information can support markedly different evaluations of victim agency, allowing individuals to selectively emphasise or downplay particular aspects of the case in ways that align with their moral intuitions.

Many of the justifications for blame attribution reflected common ST misconceptions identified from past research. For instance, previously identified myths that downplay the difficulties in leaving abusers (Houston-Kolnik et al., 2016; Mojtahedi et al., 2025) and the inability to recognise that an individual violating the law can also be a victim of trafficking (Birks and Gardner, 2019; Buckley, 2009; Santana, 2018) were frequently brought up. Victim-blaming narratives ultimately demonstrated attempts at explaining the victim’s ordeal as a product of past choices (e.g., drug use), echoing prior observations around the role of Just World Beliefs (JWB) in promoting victim-blaming within sexual violence (Hayes et al., 2013; Russell and Hand, 2017). However, the presence of JWB was not objectively measured in the present study.

### 4.2 Conditional empathy around the “deserving victim”

The findings suggest that empathetic concern was often conditional rather than absolute, with participants differing in the extent to which they viewed the victim as deserving of support and absolution from responsibility. This conditional empathy appeared to be influenced by how closely the victim was perceived to meet certain criteria associated with legitimate victimhood, such as whether she was viewed as having a genuine choice in the sexual activity, whether coercion was evident, and whether she was perceived as morally innocent outside of the exploitation. Particularly, the victim’s drug use was used to undermine their legitimacy by some, this corresponds with past research demonstrating that many individuals fail to understand how an individual breaking the law can simultaneously be a victim (Birks and Gardner, 2019; Buckley, 2009; Santana, 2018). Moreover, participants appeared to differ in their degree of empathetic concern, as reflected by categorisations of empathy, empathetic but critical and non-empathetic. Importantly, this pattern was evident both across participants, who frequently drew contrasting conclusions from the same case details, and within individual participants, many of whom simultaneously expressed concern for the victim whilst attributing some degree of responsibility for her exploitation. Thus, it is possible that participants construct a hierarchy of victimhood in which the degree of empathy is dependent on the perceived legitimacy of the victim. Taken together, the present findings suggest that empathy and blame often coexist, with participants calibrating the extent of their support according to perceived victim legitimacy. This corresponds with previously established myths around the “ideal victim,” the notion of a rigid stereotyped criteria that a victim must fit to be considered legitimated and deserving of empathy and justice (Vonderhaar and Carmody, 2015; Du Mont et al., 2003; Venema, 2016).

### 4.3 Understanding the complexity of coercive control

The vignette used in the present study involved a clear case of intimate partner coercive control, whilst this is not generalisable to all cases of sexual exploitation, it is a common element in many cases with perpetrators either exploiting women that they had pre-existing relationships with or feigning intimacy with acquired victims (Barter et al., 2009). Within such cases, the victim’s bond to the perpetrator is used to facilitate compliance and control more easily. Many empathetic justifications acknowledged the complexity of intimate partner abuse, this included acknowledging the psychological and emotional factors that can obfuscate an individual’s awareness of intimate partner abuse and make dissent or escape difficult. These challenges were not appreciated within victim-blaming responses, instead, such participants reduced the complexity of the coercive control down to behaviour that could have been rejected by the victim. Accordingly, many victim-blaming responses downplayed the influence of psychological manipulation and insisted that the victim’s compliance stemmed from weakness. Whilst the present study design does not permit us to establish the direction of the relationship between coercive control knowledge and victim empathy, past research demonstrates that limited knowledge around the complexity of ST (Mojtahedi et al., 2026) or victimisation more generally (Fox and Cook, 2011) can cause individuals to respond to victims with reduced empathy and greater victim-blaming. This opens up the discussion on whether educational interventions can offer some practical utility in reducing victim-blaming and improving empathetic responses to victims. This would be of particular relevance to victim-facing professions. Whilst the present study only examined the attitudes of lay persons, past research has demonstrated similar patterns of unempathetic and victim-blaming attitudes among medical and policing professionals responding to sexual violence (Gill and Anitha, 2024; Rajaram and Tidball, 2018). Moreover, research has demonstrated that medical and legal professionals who receive targeted training around victim groups demonstrate greater awareness around victims and improvement in their professional practice (see Beck et al., 2015; Farrell and Pfeffer, 2014). While fact-based intervention studies have demonstrated mixed effects in reducing ST victim-blaming (e.g., Miller et al., 2023; Mojtahedi et al., 2025), such studies have focused on dispelling ST myths rather than educating participants about the interpersonal complexities of coercion and control. Integrating a more comprehensive educational exercise within the AEAM framework (i.e., addressing misconceptions around controllability and internal/external influence) may enhance impact by directly challenging attributional drivers of blame.

### 4.4 Credibility-based scepticism

What became apparent from participants’ narratives was that evaluations of the victim were informed not only by the details of the case but also by perceptions of the victim’s credibility and legitimacy. Whilst many participants accepted the victim’s account and responded with empathy and support, others questioned the extent of her victimisation, her motivations, or the completeness of the information presented. This aligns with Epstein and Goodman (2019), who argue that the accounts of survivors of intimate partner violence are frequently subject to a “credibility discount,” whereby observers evaluate testimony through pre-existing assumptions about how a genuine victim should behave, respond, or present their experiences.

Several participants also suggested that the perpetrator’s account would be required before an informed judgement regarding victim accountability could be made. Such comments reflected doubts about the victim’s account and, in some cases, implied that her gender influenced perceptions of her credibility, thereby questioning the authenticity of her victim status. This finding aligns with Epstein and Goodman's (2019) concept of a “hermeneutics of suspicion,” whereby individuals focus on missing information, alternative explanations, or mitigating factors before accepting a victim’s account as credible. Taken together, these responses suggest that assessments of credibility played an important role in shaping participants’ interpretations of the scenario.

Whilst approaching criminal cases with a sense of neutrality towards both parties involved can be seen as objective and reliable, especially within the context of court cases, the fact that some participants were unable to determine empathetic concern towards a victim despite a comprehensive account of abuse suggests a lack of victim believability. This corresponds with juror research demonstrating that complainant believability is often questioned most notably in cases of sexual violence (Devine and Mojtahedi, 2021; Richardson et al., 2025), this is often informed by typical “she lied” sexual violence myths as well as “ideal victim” expectations, as well as more inherent biases towards victim groups, including hostile sexism (Lilley et al., 2023; Mojtahedi et al., 2026).

### 4.5 Gender differences responses towards the victim

Although the present study was not designed to examine gender differences, the exploratory comparisons revealed that the small number of explicitly non-empathetic justifications were provided by female participants. This contrasts with previous research suggesting that women typically express greater empathy and more supportive attitudes towards victims of sexual violence than men, often attributed to higher empathic capacity and greater identification with victims (Grubb and Harrower, 2008; Osman, 2011). One possible explanation is defensive attribution theory, whereby women may attribute greater responsibility to victims as a means of reducing their own perceived vulnerability to victimisation (see Pinciotti and Orcutt, 2020). However, this interpretation remains speculative, particularly given the very small number of non-empathetic responses and the absence of meaningful gender differences in victim-blaming attributions more broadly. Nevertheless, these findings reinforce the broader conclusion that unempathetic and victim-blaming responses are not confined to the opposite sex. Rather, they may emerge across individuals with differing backgrounds, further illustrating the complexity of public evaluations of ST victims and supporting the present finding that empathic concern and blame attribution can coexist within the same individual.

### 4.6 Limitations and directions for future research

Several limitations should be considered when interpreting these findings. A caveat of the qualitative design of the present study was that it did not permit direct measurement of empathic responses or causal inference regarding the relationship between ST attitudes and victim evaluations. Consequently, it remains unclear whether participants’ narratives reflect underlying attitudes that actively shape their judgements, or whether they function as post-hoc rationalisations of more intuitive or ideologically driven responses (e.g., sexism; Mojtahedi et al., 2026). This distinction is particularly relevant in light of the social intuitionist model (Haidt, 2001), which posits that moral judgements are often driven by rapid, affective intuitions that are subsequently justified through reasoning. It is therefore plausible that participants selectively drew upon particular narrative elements (e.g., grooming, choice, vulnerability) to support pre-existing moral positions, rather than constructing judgements through deliberative evaluation of the case. Further experimental research is needed to determine causal pathways.

Data were collected via online surveys, limiting narrative depth compared to interviews. Future studies should employ in-person designs to capture richer reasoning processes and allow for more complex qualitative analysis. Second, reliance on a single vignette constrains generalisability of our interpretations to the wider breadth of ST cases which are heterogeneous in nature; reliance on UK-only responses also limits the generalisability to the wider population (with research demonstrating some key differences in ST victim attitudes between US and UK participants, see Mojtahedi et al., 2026). As such it is questionable how consistent the observed themes would be within distinct cases (e.g., where the abuser is not a partner); replication with diverse cases and populations is therefore needed. While the current study corresponds with the AEAM by demonstrating that locus of control and controllability attributions are key themes within empathetic reactions, future research should employ more rigorous experimental designs to deepen understanding of victim judgements within this framework. Specifically, studies could manipulate attributional elements across multiple vignettes and incorporate a broader range of empathy measures—such as physiological and behavioural indicators alongside self-reported assessments. This approach would enable the development of a more comprehensive model of the cognitive and affective processes underlying responses to ST cases.

It is also worth considering the possibility that the adoption of the AEAM in the academic literature may have indirectly influenced the results of the content analysis. The inductive nature of the latter is maintained, as the coders did not use the AEAM as guidance for the coding; one of them is based in an altogether different field of research and had never encountered this model before the content analysis was finished. However, it is reasonable to consider the possibility that one of the coders may have encountered the AEAM before and may have been unconsciously influenced by it while conducting the content analysis of the source material.

An additional limitation of the survey design was identified following data collection. To explore empathic responses, participants were asked to justify whether or not they felt empathy towards the victim. To assist participants in formulating their response, an example statement was provided within the question (“I [feel/do not feel] sorry/empathy for Stephanie because…”). Although intended as a simple prompt, the inclusion of both “empathy” and “feeling sorry” may have encouraged participants to draw upon a broader range of affective responses when answering the question. Furthermore, participants were not provided with a formal definition of empathy, leaving scope for individual interpretation of the construct. Consequently, some responses may have reflected related affective reactions, such as sympathy, pity, compassion, or general concern for the victim, rather than empathy in its strict psychological sense. While this limitation does not affect the identification of the narrative themes underpinning participants’ responses, it does introduce some ambiguity regarding the precise form of affective response being justified.

In addition to building on the current study’s methodological shortcomings, an avenue for future research concerns the implications of varying degrees of victim-blaming. Although victim-blaming is generally considered harmful because of its potential to undermine support for victims, there is a lack of insight on how differing degrees or forms of victim-blaming can impact victims and criminal justice outcomes. Future research should therefore examine whether there are meaningful thresholds at which blame attribution begins to influence helping behaviour, credibility assessments, or willingness to hold perpetrators accountable.

## 5 Conclusion

This study provides novel insight into the cognitive and affective processes underpinning evaluations of ST victims. Whilst several theoretical perspectives helped contextualise the findings, participants’ narratives consistently centred on perceptions of agency, preventability, and choice. These factors emerged across both empathic and victim-blaming responses, suggesting that attributional reasoning may provide a particularly useful framework for understanding how individuals construct evaluations of ST victims. Drawing on lay perspectives, the findings demonstrate that empathy and victim-blaming are not mutually exclusive. Rather than reflecting distinct groups of supporters and blamers, many participants simultaneously expressed concern for the victim whilst attributing some degree of responsibility for her exploitation. These findings suggest that support for victims is often conditional and influenced by perceptions of legitimacy, agency, and coercion.

## Data Availability

The raw data supporting the conclusion can be made available upon request from the corresponding author.
