# Supplementary material for: Understanding how people rationalize victim-blaming and empathy in sex trafficking: a qualitative analysis of 130 narratives
Source: Front Psychol. 2026 Aug 5;17:1773545. doi: 10.3389/fpsyg.2026.1773545 (PMC13485739; doi:10.3389/fpsyg.2026.1773545)
Supplement: Supplementary file 1 [file Supplementary_file_1.docx]

**You are now asked to read the following case and answer multiple questions relating to your thoughts on the events described. There will be some additional questions included to make sure you have read and understood the case.**

**The Case:** 

Stephanie (23 years of age) has accused her former partner, Ryan (38) of sexually exploiting her for financial gain. According to Stephanie, the exploitation started 4 months into their relationship and lasted for almost a year until Stephanie ran away from Ryan and reported the incidents to the police. Ryan is currently under police investigation. Below are transcript extracts taken from an Interview with Stephanie about how she initially met Ryan and also about the alleged exploitation.

**How Stephanie met Ryan:**
“I first met Ryan at a pub called ‘The Drunk Duck’, I was around 15 at the time. I would run away from home now and again because my home wasn’t nice, my parents were always drunk and it was just a shit place to live. The Drunk Duck didn’t ID me so I would sometimes go there, I was broke but some of the locals would buy me drinks. Ryan was one of these locals, he was a nice guy and we would have genuine conversations about things silly thing. He would also get me cocaine which we would have together. We were just friends though, he never tried to kiss me or touch me at that time. I didn’t see him for like five years after that, turns out he moved to another city, but then I saw him again when I was around 20 and we hit it off. I ended up going back to his place one night to have sex and after that, we started seeing each other a lot more. I ended up moving into his house. He made me feel so loved, buying me gifts and saying really sweet things. No one had ever treated me like that before”.

**How the alleged exploitation started:**
“A few months later, we were madly in love. Me and Ryan would have ‘date nights’ on Saturdays, which was basically a trip to a bar or pub to get drunk, sometimes we would take cocaine but It was always Ryan’s idea. He would always have a bag of the stuff and he would tell me to do some with him. On one of these nights, we were at a bar and it was midnight. One of Ryans so called ‘friends’ had told him he wanted to have sex with me and offered to pay Ryan £60. Ryan asked me to go to the toilets with him and have sex with him. I thought he was joking at first and laughed but then he told me that we needed the money and made me feel bad because he had paid for our drinks all night. I got angry and said ‘absolutely not!’ but he kept on persisting,  he kept asking me to do him this favour just once. He continued to hassle me for thirty minutes, it made very uncomfortable. He didn’t become aggressive or threaten me but it was clear that he wasn’t going to let this go so I eventually agreed and had sex with his friend.
The next day I was disgusted in myself, but Ryan was so nice to me. He apologised and spent the whole day cuddling me and trying to cheer me up. He told me that we needed the money because he could no longer support us with his job at the Car Garage. He made it clear that I would have to occasionally help out by making more money. I already had a supermarket job but the pay was rubbish and not enough to support us. I still didn’t want to do it but he made me feel so guilty and his constant persistence started to frighten me. He was no longer asking ‘if’ I would do it again and started telling me that I would be doing it again. The next time he made me sleep with someone was the week after, he told me that someone he  knew had offered us £100 to sleep with me. He told me he loved me and that he only wanted me to do this so that we could have a better life. I said no but he kept pressuring me and I knew he wouldn’t stop so I reluctantly agreed and went back to this man’s house for sex. I was in a difficult place because I loved Ryan and would do anything for him but I really didn’t want to do this. Soon there were multiple men lined up for me to sleep with each week. He became more forceful as well. I told him several times that I didn’t want to do this anymore,  but he would shout at me, he threatened to kick me out of the house and told me that he was the only person who cared about me. I felt scared and alone, so I just did what he told me to do. I know it sounds stupid but I still felt that he loved me because he would always be so romantic during the day. I started taking more cocaine before having sex with men, it was the only way I could cope with the trauma. Ryan always had cocaine on him and would give me some, but only if I had sex with the men.”

**On leaving Ryan and reporting the incident to the police:**
 “I wanted to get out but I didn’t know who to turn to. I didn’t really have any close friends because Ryan didn’t like me spending too much time with other people. He would accompany me whenever I wanted to go somewhere. I felt that if he ever caught me trying to escape he would punish me badly so I was scared to try and leave. But eventually I had enough,  and ran away from the house when he was out. It was a hard thing to do because I still loved him and believed that he loved me. At the same time, I was terrified that he would come looking for me so I went straight to a women’s shelter and asked for advice. They told me to speak to the police which I did and since then, Ryan has been arrested and I have moved into a temporary hostel.”
